# The Medial Prefrontal Cortex, Nucleus Accumbens, Basolateral Amygdala, and Hippocampus Regulate the Amelioration of Environmental Enrichment and Cue in Fear Behavior in the Animal Model of PTSD

**DOI:** 10.1155/2022/7331714

**Published:** 2022-02-07

**Authors:** Ying Hao Yu, Yeou San Lim, Chen Yin Ou, Kai Chieh Chang, Arthur C. Tsai, Fang Chih Chang, Andrew Chih Wei Huang

**Affiliations:** ^1^Department of Psychology, Fo Guang University, Yilan County 26247, Taiwan; ^2^Department of Biotechnology and Animal Science, National Ilan University, Yilan County 26247, Taiwan; ^3^Institute of Statistical Science, Academia Sinica, Taipei, Taiwan

## Abstract

A growing body of evidence showed that environmental enrichment (EE) ameliorated footshock-induced fear behavior of posttraumatic stress disorder (PTSD). However, no research comprehensively tested the effect of EE, cue, and the combination of EE and cue in footshock-induced fear behavior of PTSD symptoms. The present study addressed this issue and examined whether the medial prefrontal cortex (mPFC, including the cingulate cortex 1 (Cg1), prelimbic cortex (PrL), and infralimbic cortex (IL)), the nucleus accumbens (NAc), the basolateral amygdala (BLA), and the hippocampus (e.g., CA1, CA3, and dentate gyrus (DG)) regulated the amelioration of the EE, cue, or the combination of EE and cue. The results showed that EE or cue could reduce fear behavior. The combination of EE and cue revealed a stronger decrease in fear behavior. The cue stimulus may play an occasion setting or a conditioned stimulus to modulate the reduction in fear behavior induced by footshock. Regarding the reduction of the EE in fear behavior, the Cg1 and IL of the mPFC and the NAc upregulated the c-Fos expression; however, the BLA downregulated the c-Fos expression. The mPFC (i.e., the Cg1, PrL, and IL) and the hippocampus (i.e., the CA1, CA3, and DG) downregulated the c-Fos expression in the suppression of the cue in fear behavior. The interaction of EE and cue in reduction of fear behavior occurred in the Cg1 and NAc for the c-Fos expression. The data of c-Fos mRNA were similar to the findings of the c-Fos protein expression. These findings related to the EE and cue modulations in fear behavior may develop a novel nonpharmacological treatment in PTSD.

## 1. Introduction

Posttraumatic stress disorder (PTSD) is a complicated mental illness [1]. PTSD individuals often encounter a variety of psychiatric symptoms that include feeling fear, helplessness, horror, increased arousal, autonomic hyperarousal activities, fear sensitization, serious startle responses, hypervigilance, insomnia, irritability, and impaired concentration [2–4]. Concerning the numerous symptoms of PTSD, the fear symptom through classical conditioning to form fear memory in the animal model of PTSD effectively simulated the PTSD's human symptoms [5]. Moreover, PTSD's fear symptom is crucial to the etiology of PTSD, and it is highly preserved in the process of evolution cross-species [5].

In light of the previous studies, environmental enrichment (EE) exposures can effectively reduce the PTSD symptoms in the animal model [6–11]. For example, EE reduced anxiety due to differentially activating the serotonin system and neuropeptide system in the PTSD animal model [11]. Chronic EE exposures reduced neonatal isolation-induced contextual freezing; however, chronic EE exposures did not affect single prolonged stress-related to anxiety behaviors or analgesia [9]. Acute EE exposures facilitated fear extinction through neuropeptide Y-Y1 receptor modulation in the hippocampus [10]. EE enhanced anxiolytic effect induced by deep-brain stimulation in the medial prefrontal cortex (mPFC) in the PTSD animal model [6]. Traumatic stress caused the reduction in the volume of the hippocampus and central amygdala, and EE was able to ameliorate trauma-induced volume decreases of the hippocampus resulted in behavioral, morphological, and molecular changes [8]. EE facilitated the negative feedback of the hypothalamus-pituitary-adrenal gland (HPA) axis-related stress system and then improved abnormal behaviors through decreasing the glucocorticoid receptors in the hypothalamus and hippocampus [7]. Therefore, EE is a nonpharmacological treatment in reducing PTSD symptoms at behavioral, neuronal, and molecular levels.

The EE manipulation combined exposure of sensory, physical, cognitive, and social stimuli in the contextual surroundings for individuals, and the EE simulated a contextual environment. Animals exposed the entire components of sensory, physical, social, and cognitive stimuli [12–14]. In contrast to the EE stimuli, the cue is a single component or stimulus of the contextual stimuli [15]. Based on the previous evidence, the different category of fear conditioning is controlled by the different neural substrates: the hippocampus is contributable to the context-induced fear conditioning [15, 16], whereas the amygdala modulates the cue-induced fear conditioning [17, 18]. The hippocampus plays a function in configural memory and the encoding process of contextual stimulus [19]. The amygdala is essential to regulate the negative valence of emotion and negative emotional processes [20]. Recently, accumulated studies have clarified the roles of the mPFC, amygdala, and hippocampus in the fear conditioning of the PTSD symptoms after encountering a traumatic stress event [21–24]. The individual who experienced traumatic events was revealed to decrease the volumes of the hippocampus and anterior cingulate cortex; however, it increased the hyperactivity of the amygdala; moreover, the mPFC showed dysfunction that cannot inhibit the activity of the amygdala [21]. The hippocampus contributed to spatial learning and declarative memory; moreover, the hippocampus was projected from the amygdala, and thereby, the hippocampus-related functions, including spatial learning and memory, were regulated by the amygdala's activity [25]. Alternatively, little research has demonstrated that the nucleus accumbens (NAc) might be involved in the PTSD symptoms associated with stress behaviors [26, 27]. Therefore, the study examined whether the mPFC, amygdala, hippocampus, and NAc mediated fear behavior of PTSD symptoms.

Altogether, some critical issues should be concerned: (a) Whether EE decreased fear symptoms of PTSD. (b) Whether a stimulus cue can reduce PTSD fear symptoms. (c) Using the c-Fos labeling and the assessment of c-Fos mRNA expression with the qRT-PCR methods, whether the subareas of mPFC (e.g., the Cg1, PrL, and IL), the subregions of the hippocampus (e.g., the CA1, CA3, and DG), the NAc, and BLA were involved in the amelioration of EE, cue, or the EE and cue combination in footshock-induced fear behavior of the PTSD symptom.

## 2. Methods and Materials

### 2.1. Animals

Fifty-six male wild-type C57BL/6 mice (approximate 25~35 g at the beginning of the experiments) were bought from National Laboratory for Animal Breeding and Research Center, Taipei, Taiwan. All mice were randomly assigned into no environmental enrichment (no EE; i.e., standard housing) and EE cages. The mice of no EE and EE groups were raised with another two mice in a colony room with constant temperature (approximately 23 ± 2°C) and light-dark cycle (light on 6 : 00-18 : 00). The EE cage is a plastic surrounding shell box, which is 33.5 cm long × 25.0 cm wide × 28.8 cm high. The standard cage is a plastic box, and its surrounding size is 30.0 cm long × 18.8 cm wide × 13.5 cm high. Food and water were provided ad libitum. The experiments were performed in compliance with the American Psychological Association ethical standards for the treatment of animals. A description of the treatment details was submitted and received approval from the Institutional Animal Care and Use Committee (IACUC) of Fo Guang University. Every effort was made to minimize the animals' suffering and the number of animals used.

### 2.2. Apparatus

#### 2.2.1. Inescapable Footshock

The inescapable footshock apparatus is a box composed of a plastic surrounding shell measuring 60 cm × 60 cm × 72 cm high. The apparatus floor is composed of metal grids (0.3 cm diameter at 0.7 cm grid intervals). On the footshock procedure, mice were placed in the footshock apparatus for 2 min. Then, mice were given a 2 mA footshock (duration, 10 seconds), and the other mice received no footshock at their home cage [28].

### 2.3. Behavioral Procedure

**Figure 1:**
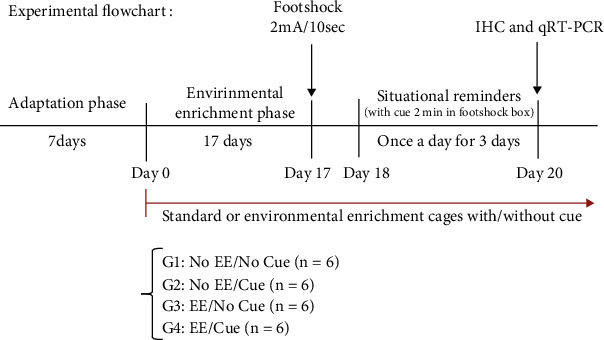
Overview of the experimental procedures. The behavioral processes were shown in the adaptation, EE phase, footshock treatment, and situational reminder phases. On days 0-17, the mice in the EE groups were subjected to the EE procedure. These mice received the EE procedure at the end of the experiment. However, the mice in the no EE groups were housed in the standard cage. On day 17, all mice were placed in the footshock box for 2 min, and then the mice were subjected to the footshock treatment (2 mA, 10 seconds). On days 18-20, all mice have given a situational reminder procedure that the mice were placed in the footshock box for 2 min with the cue ball but not any footshock. One hundred twenty minutes after the last behavioral test, the immunohistochemical staining with the c-Fos protein expression on day 20. All mice were assigned to the no EE/no cue, no EE/cue, EE/no cue, and EE/cue groups (*n* = 6, per group). EE: environmental enrichment.

All mice received an adaptation regimen for ad libitum food and water for seven days in the adaptation phase. After that, all mice were dependent on their assigned groups, and they were, respectively, raised in a mixed environment, including any combinations of no EE, EE, no cue, and cue until the end of the experiment (day 0-day 20). On day 17, all mice were given a single footshock (2 mA, 10 seconds) in the footshock apparatus. On days 18-20, all mice received the situational reminder procedure. Note, the current rate of footshock (2 mA, 10 seconds) and three freezing-times in situational reminders were determined by our previous studies [22, 29]. The mice were placed in the footshock apparatus for 2 min without footshock. One hundred twenty minutes after completing the last behavioral test, the immunohistochemical staining and the qRT-PCR method were, respectively, performed to measure c-Fos protein and c-Fos mRNA expression in the selected brain areas (see Figure 1).

All of the mice were assigned into EE or cue environments into four groups: no EE/no Cue, no EE/cue, EE/no cue, and EE/cue groups (*n* = 6, per group). Regarding the housing condition, the mice in the no EE/No cue group were given no EE and no cue in the standard cage. The no EE/cue group mice were given no EE with a cue stimulus in the standard cage. The mice in the EE/no cue group were given an EE procedure without a cue stimulus. The mice in the EE/cue group were given an EE procedure with a cue stimulus. The EE cage included two large wooden blocks, a shelter, a retreat, a tunnel, crow ball, motor running wheel, and bone toys, and the EE procedure was followed the previous study [30]. The cue stimulus was designed for the mice exposed to a plastic round ball (diameter is 3 cm) in the EE or standard cages for 24 hours. This cue stimulus, a white round ball, is different in size and texture from the crow ball of the EE cage.

### 2.4. Immunohistochemical Staining

Mice were sacrificed by sodium pentobarbital overdose after completing behavioral tests for 120 min (i.e., labeling IHC c-Fos for the best expression). When completely unresponsive, mice were perfused with 0.9% NaCl followed by 4% paraformaldehyde in 0.1 M sodium phosphate-buffered saline (PBS). The brain tissues were dissected, blocked, postfixed for 3 days, and transferred to 30% sucrose for cryoprotection for 2 days until the specimens sink to the bottom of the solution. Forty-micron coronal sections were cut on freezing using a sliding microtome. All brain slices were processed by c-Fos immunoreactivity staining. Free-floating brain slices were washed once for 10 mins in 0.1 M PBS, permeabilized in 3% H_2_O_2_ for 1 hour, washed three times in PBS for 10mins, and then soaked in normal goat serum for 1 hour. After washing with PBS once for 10 mins, the slices were incubated overnight with the specific first antibody (i.e., sheep anti-c-Fos primary antibody at a dilution of 1 : 500). This antibody was raised against a peptide sequence to enable crossreaction with other proteins for detecting c-Fos immunoreactivity. The slices were then washed once with PBS for 10 mins, and then, these slices were incubated in a second antibody (i.e., biotinylated anti-sheep secondary antibody at a dilution of 1 : 500) for 2 hours. Ten minutes with PBS washing later, the bound secondary antibody was amplified using the Vector Elite ABC kit.

The positive expression nucleus of neurons was quantified for the selected brain areas. Counting was performed visually at ×20 magnification by a researcher blinded to the condition of each mouse. Every third slice of the brain tissue was selected into an available counted section. The software ImageJ was used to count the amounts of c-Fos-positive neurons [31].

### 2.5. Real-Time Quantitative PCR of c-Fos

Based on the manufacturer's instructions, total RNA was extracted with TRIzol Reagent (Invitrogen, Carlsbad, CA, USA). For cDNA synthesis, total RNA was applied with random hexamers. Later, the reverse-transcription PCR was the amplification of *c-fos*. For the amplification, it was initiated with a pair of *c-fos* primers (forward: 5′-TCCACTGCCTGGGACAGAA-3′; reverse: 5′-CGCAGCGATCTTCATCAAAC-3′) with a volume of 20 *μ*l. For undergoing the process, it was a denaturation stage at 95°C for 10 minutes. After that, 28 cycles of denaturation were conducted at 95°C for one minute. The primer was annealing at 55°C for 30 seconds and extension at 72°C for 45 seconds. The cycling steps were completed, and the final extension was at 72°C for five minutes. The reactions were repeated three times and were performed in an ABI PRISM 7500 Sequence Detection System (Applied Biosystems, Thermo Fisher Scientific, USA). The mean expression levels of the housekeeping gene were with a pair of beta-actin primers (forward: 5′CAACTTGATGTATGAAGGCTTTGGT-3′; reverse: 5′-ACTTTTATTGGTCTCAAGTCAGTGTACAG-3′). It was used as the internal control to normalize the variability of *c-fos* expression levels. Relative changes in the gene expression were measured using the 2^-*ΔΔ*CT^ method [32].

### 2.6. Statistical Analysis

A three-way mixed (environmental enrichment vs. cue vs. session) analysis of variance (ANOVA) was performed for the freezing time. One-way ANOVA was conducted for the total freezing time in the main effect of environmental enrichment, cue, and situational reminders. Furthermore, two-way mixed (group vs. session) ANOVA was performed for the freezing time over sessions 1-3 in situational reminders and total freezing time among the no EE/no cue, no EE/cue, EE/no cue, and EE/Cue groups. When appropriate, the post hoc with Tukey's Honest Significant Difference (HSD) test was performed.

Regarding the examination of the c-Fos protein expression and c-Fos mRNA expression, two-way ANOVA analysis was conducted, and one-way ANOVA analysis was performed. For c-Fos protein expression analysis, the selected brain areas were determined, including Cg1, PrL, IL, NAc, BLA, CA1, CA2, and DG. For c-Fos mRNA expression analysis, the mPFC, NAc, amygdala, and hippocampus were determined. When appropriate, the post hoc with Tukey's HSD test was performed. ^∗^*p* < 0.05 was considered statistically significant compared to the no EE/no cue group. #*p* < 0.05 was considered statistically significant compared to the no EE/cue group. $*p* < 0.05 was considered statistically significant compared to the EE/no cue group.

## 3. Results

### 3.1. PTSD Behavioral Tests

**Figure 2:**
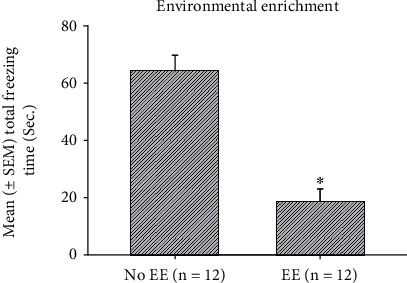
Assessments of the mean (± SEM) total freezing time (Sec.) for the min effect of EE in the no EE (*n* = 12) and EE (*n* = 12) conditions. EE: environmental enrichment. ^∗^*p* < 0.05 indicates significant differences compared to the no EE condition.

**Figure 3:**
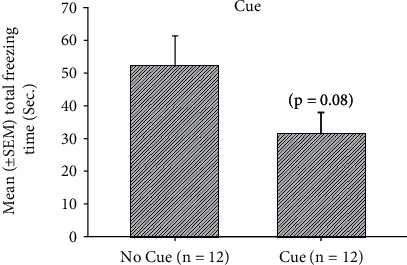
Assessments of mean (± SEM) total freezing time (Sec.) for the main effect of the cue in the no cue (*n* = 12) and cue (*n* = 12) conditions. ^∗^*p* < 0.05 indicates significant differences compared to the no cue condition.

**Figure 4:**
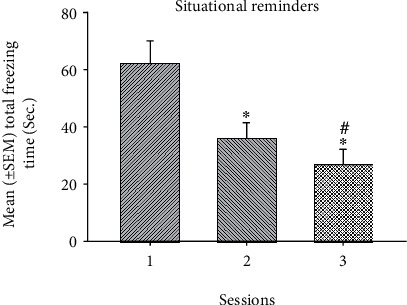
The mean (± SEM) total freezing time (Sec.) in the situational reminders for sessions 1-3. ^∗^*p* < 0.05 indicates significant differences compared to session 1. #*p* < 0.05 indicates significant differences compared to session 2.

To examine the effect of environmental enrichment and cue in freezing behavior of the PTSD animal model, a 2 × 2 × 3 three-way mixed ANOVA analysis indicated that significant differences occurred in the factor of EE (*F* (1, 20) = 70.73, *p* < 0.05), cue (*F* (1, 20) = 14.14, *p* < 0.05), and session (*F* (2, 40) = 27.95, *p* < 0.05). Nonsignificant differences occurred in the interaction of EE and cue (*F* (1, 20) = 3.24, *p* > 0.05), the interaction of EE and session (*F* (2, 40) = 2.11, *p* > 0.05), the interaction of cue and session (*F* (2, 40) = 0.28, *p* > 0.05), and the interaction of EE, cue, and session (*F* (2, 40) = 0.89, *p* > 0.05). The results indicated that EE, cue, and session had significant differences (Table 1). Furthermore, one-way ANOVA analysis was performed. The results showed that the main effect of EE was significantly decreased in total freezing time (*F* (1, 22) = 41.62, *p* < 0.05; Figure 2). The main effect of cue was seemingly decreased in total freezing time (*F* (1, 22) = 3.21, *p* = 0.08; Figure 3). The main effect of session was significantly decreased from session 1 to session 3 (*F* (2, 46) = 27.61, *p* < 0.05; Figure 4). The post hoc Tukey's HSD appeared that total freezing time of session 2 and 3 was significantly decreased when compared to session 1 (*p* < 0.05). The total freezing time of session 3 was significantly decreased than that of session 2 (*p* < 0.05). In summary, total freezing time was significantly decreased as manipulations of EE, cue, and sessions.

**Figure 5:**
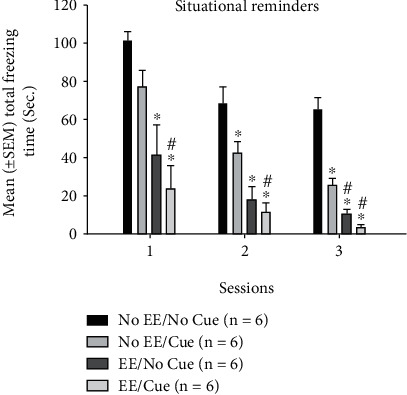
Mean (± SEM) freezing time for the situational reminder in sessions 1-3 for the no EE/no cue, no EE/cue, EE/no cue, and EE/cue groups (*n* = 6, per group). EE: environmental enrichment. ^∗^*p* < 0.05 indicates significant differences compared to the no EE/no cue group; #*p* < 0.05 indicates significant differences compared to the no EE/cue group.

Alternatively, a 4 × 3 mixed two-way ANOVA analysis was conducted for freezing behavior. The results showed that significant differences occurred in the factor of group (*F* (3, 20) = 29.37, *p* < 0.05) and session (*F* (2, 40) = 27.95, *p* < 0.05). However, there was a nonsignificant difference in the interaction of group and session (*F* (6, 40) = 1.09, *p* > 0.05). Post hoc with Tukey's HSD tests showed that the freezing time in the no EE/cue, EE/no cue, and EE/cue groups was seemingly decreased compared to the no EE/no cue group in sessions 1 and 2 (*p* < 0.05). In session 3, the freezing time in the no EE/cue, EE/no cue, and EE/cue groups was significantly decreased than that of the no EE/no cue group (*p* < 0.05); moreover, the EE/no cue and EE/cue groups were significantly decreased compared to the no EE/cue group (*p* < 0.05). Therefore, the combination of EE and cue exhibited the lowest freezing time. EE or cue manipulations could suppress freezing behavior. The EE manipulation likely reduced much more freezing time when compared to the cue manipulation (Figure 5).

**Figure 6:**
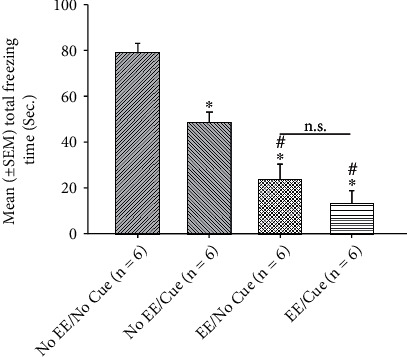
Mean (± SEM) total freezing time from the merged data in sessions 1-3 for the no EE/no cue, no EE/cue, EE/no cue, and EE/cue groups (*n* = 6, per group). EE: environmental enrichment. ^∗^*p* < 0.05 indicates significant differences compared to the no EE/no cue group; #*p* < 0.05 indicates significant differences compared to the no EE/cue group. (n.s.): nonsignificant differences.

To analyze mean (± SEM) total freezing time that was merged with the behavioral data overall sessions 1-3, two-way ANOVA was conducted to show that significant differences occurred in the factor of EE (*F* (1, 20) = 70.73, *p* < 0.05), cue (*F* (1, 20) = 14.14, *p* < 0.05), and the interaction of EE and cue (*F* (1, 20) = 3.25, *p* < 0.05). Furthermore, one-way ANOVA indicated that the factor of group was significant differences (*F* (3, 20) = 29.37, *p* < 0.05). Post hoc Tukey's HSD tests showed that the no EE/cue, EE/no cue, and EE/cue were significantly decreased in total freezing time compared to the no EE/no cue group (*p* < 0.05). The total freezing time of the EE/no cue and EE/cue group was significantly decreased than that of the no EE/cue group (*p* < 0.05). However, the freezing time of the EE/no cue group was not significantly different from that of the EE/cue group (*p* > 0.05; Figure 6). The similar evidence as mentioned above that the EE manipulation might be effective to reduce total freezing time. The combination of EE and cue treatments was likely the greatest reduction in total freezing behavior.

### 3.2. Immunohistochemical Staining with c-Fos Protein Expression

To examine the involvements of selected brain areas in the EE, cue, or the interaction of EE and cue, a 2 × 2 two-way ANOVA was conducted for the c-Fos expression after testing freezing behavior. The results of the EE manipulation showed that the c-Fos expression was significant increases in Cg1, IL, and NAc but decreases in BLA compared to the no EE manipulation (*p* < 0.05); the nonsignificant c-Fos expression occurred in the PrL, CA1, CA3, and DG (*p* > 0.05). The cue manipulations showed that the c-Fos expression was significant decreases in the Cg1, PrL, IL, CA1, CA3, and DG compared to the no cue manipulation (*p* < 0.05); nonsignificant differences occurred in the NAc and BLA (*p* > 0.05). The EE and cue interactions were significant differences in the C1g and NAc (*p* < 0.05); however, the other brain areas did not show significant differences (*p* > 0.05; Table 2).

**Figure 7:**
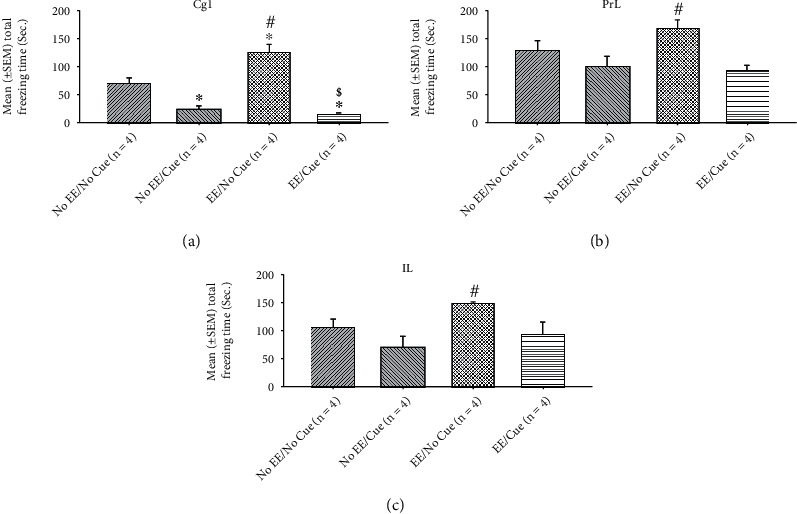
Mean (± SEM) c-Fos expression in the Cg1 (a), PrL (b), and IL (c) for the no EE/no cue (*n* = 6), no EE/cue (*n* = 6), EE/no cue (*n* = 6), and EE/cue groups (*n* = 6). EE: environmental enrichment. ^∗^*p* < 0.05 indicates significant differences compared to the no EE/no cue group; #*p* < 0.05 indicates significant differences compared to the no EE/cue group.

On the other hand, one-way ANOVA was conducted to analyze the c-Fos expression in the selected brain areas. The results showed that a significant difference occurred in the factor of the group (*F*(3, 12) = 29.35, *p* < 0.05). In post hoc Tukey's HSD tests, the Cg1 showed that the EE/no cue was significantly increased in the c-Fos expression than the no EE/no cue and no EE/cue groups (*p* < 0.05); the no EE/cue and EE/cue were significantly decreased in the c-Fos expression compared to the no EE/no cue group (*p* < 0.05); the EE/cue was significantly decreased in the c-Fos expression than the EE/cue group (*p* < 0.05; Figure 7(a)).

In the PrL c-Fos expression measurements, a significant difference occurred in the factor of group (*F*(3, 12) = 5.21, *p* < 0.05). In post hoc Tukey's HSD tests, the results showed that the EE/no cue was significantly increased in the c-Fos expression than the no EE/cue group (*p* < 0.05; Figure 7(b)).

**Figure 8:**
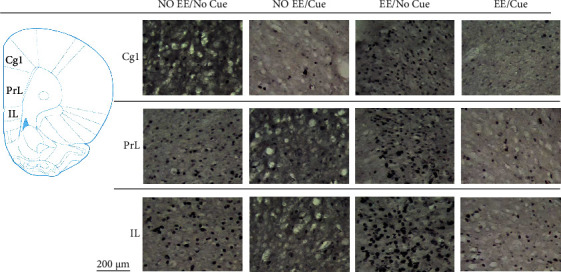
(a) A schematic brain atlas for the Cg1, PrL, and IL of the mPFC. (b) Representative photomicrographs of the c-Fos expression for the Cg1, PrL, and IL in the no EE/no cue, no EE/cue, EE/no cue, and EE/cue groups (*n* = 4, per group). Scale bar represents 200 *μ*m.

In the IL c-Fos expression measurements, a significant difference occurred in the factor of group (*F* (3, 12) = 4.44, *p* < 0.05). In post hoc Tukey's HSD tests, the results showed that the EE/no cue was significantly increased in the c-Fos expression than the no EE/cue group (*p* < 0.05; Figures 7(c) and 8).

**Figure 9:**
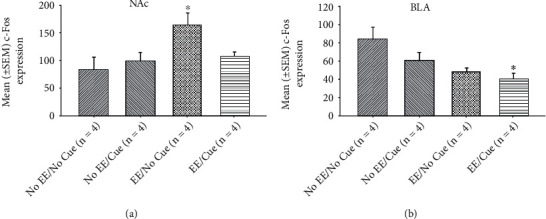
Mean (± SEM) c-Fos expression in the NAc (a) and BLA (b) for the no EE/no cue (*n* = 6), no EE/cue (*n* = 6), EE/no cue (*n* = 6), and EE/cue groups (*n* = 6). EE: environmental enrichment. ^∗^*p* < 0.05 indicates significant differences compared to the no EE/no cue group; #*p* < 0.05 indicates significant differences compared to the no EE/cue group.

In the NAc c-Fos expression measurements, a significant difference occurred in the factor of group (*F* (3, 12) = 4.18, *p* < 0.05). In post hoc Tukey's HSD tests, the results showed that the EE/no cue was significantly increased in the c-Fos expression than the no EE/no cue group (*p* < 0.05; Figure 9(a)).

**Figure 10:**
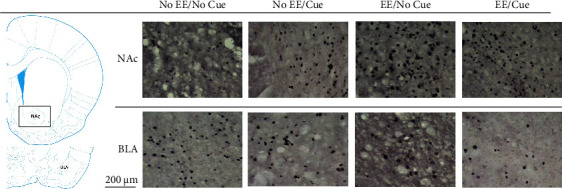
(a) A schematic brain atlas for the NAc and the amygdala's BLA. (b) Representative photomicrographs of the c-Fos expression for the NAc and the amygdala's BLA in the no EE/no cue, no EE/cue, EE/no cue, and EE/cue groups (*n* = 4, per group). Scale bar represents 200 *μ*m.

In the BLA c-Fos expression measurements, a significant difference occurred in the factor of the group (*F* (3, 12) = 4.32, *p* < 0.05). In post hoc Tukey's HSD tests, the results showed that the EE/cue was significantly decreased in the c-Fos expression than the No EE/no cue group (*p* < 0.05; Figures 9(b) and 10).

**Figure 11:**
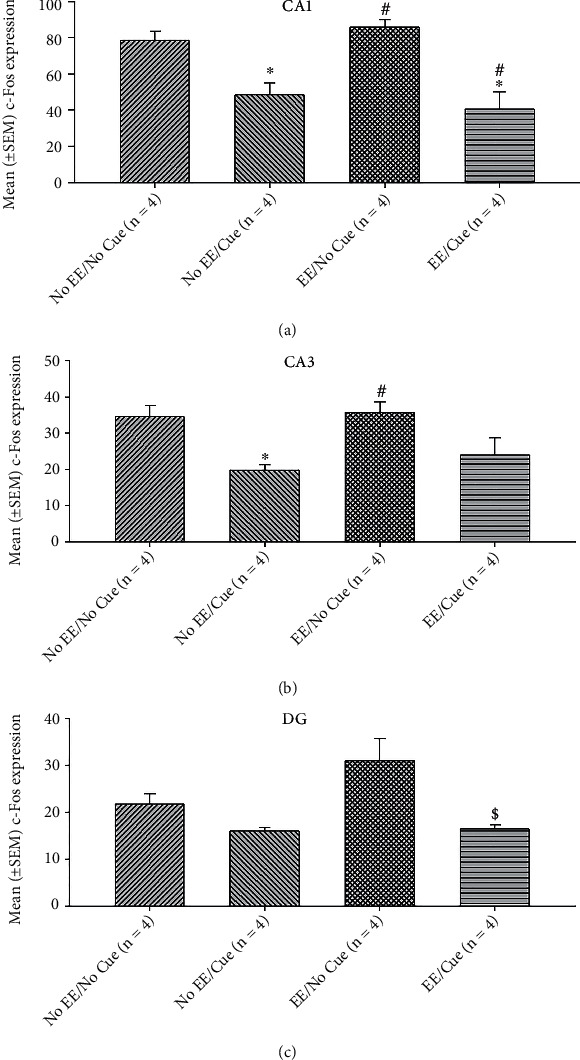
Mean (± SEM) c-Fos expression in the CA1 (a), CA3 (b), and DG (B) for the no EE/no cue (*n* = 6), no EE/cue (*n* = 6), EE/no cue (*n* = 6), and EE/cue groups (*n* = 6). EE: environmental enrichment. ^∗^*p* < 0.05 indicates significant differences compared to the no EE/no cue group; #*p* < 0.05 indicates significant differences compared to the no EE/cue group.

In the CA1 c-Fos expression assessments, a significant difference occurred in the factor of group (*F* (3, 12) = 11.40, *p* < 0.05). In post hoc Tukey's HSD tests, the results showed that the no EE/cue and EE/cue groups were significantly decreased in the c-Fos expression than the no EE/no cue group (*p* < 0.05); the EE/no cue and EE/cue were significantly increased and decreased in the c-Fos expression, respectively, compared to the no EE/cue group (*p* < 0.05; Figure 11(a)).

In the CA3 c-Fos expression assessments, a significant difference occurred in the factor of group (*F* (3, 12) = 5.81, *p* < 0.05). In post hoc Tukey's HSD tests, the results showed that the no EE/cue group was significantly decreased in the c-Fos expression than the no EE/no cue group (*p* < 0.05); the EE/no cue was significantly increased in the c-Fos expression compared to the no EE/cue group (*p* < 0.05; Figure 11(b)).

**Figure 12:**
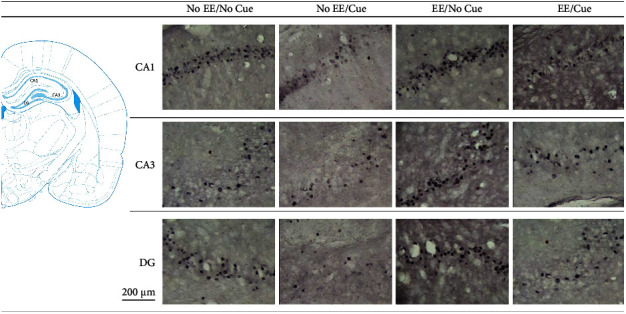
(a) A schematic brain atlas for the Cg1, CA3, and DG of the hippocampus. (b) Representative photomicrographs of the c-Fos expression for the CA1, CA3, and DG of the hippocampus in the no EE/no cue, no EE/cue, EE/no cue, and EE/cue groups (*n* = 4, per group). Scale bar represents 200 *μ*m.

In the DG c-Fos expression assessments, a significant difference occurred in the factor of group (*F* (3, 12) = 6.89, *p* < 0.05]. In post hoc Tukey's HSD tests, the results showed that the EE/no cue group was significantly increased in the c-Fos expression than the no EE/cue group (*p* < 0.05); the EE/cue was significantly decreased in the c-Fos expression compared to the EE/no cue group (*p* < 0.05; Figures 11(c) and 12).

Therefore, the Cg1, IL, and NAc were upregulated in the c-Fos expression, but the BLA was a downregulation in the c-Fos expression through the EE manipulations. The Cg1, PrL, IL, CA1, CA3, and DG were a downregulation in the c-Fos expression via the cue manipulations. Notably, the Cg1 and NAc were significant differences in the c-Fos expression under the interaction between EE and cue.

### 3.3. qRT-PCR Assessment for the c-Fos mRNA Expression

To test the c-Fos mRNA expression of EE, cue, and EE×cue in the mPFC, NAc, amygdala, and hippocampus, a 2 × 2 two-way ANOVA was conducted after testing freezing behavior. The results showed that EE manipulations were significant increases in the mPFC (*F* (1, 12) = 4.68, *p* < 0.05) and NAc (*F* (1, 12) = 14.01, *p* < 0.05); however, there was significant decreases in the amygdala (*F* (1, 12) = 10.98, *p* < 0.05) compared to the no EE manipulations. There were nonsignificant differences between the EE and no EE manipulations in the hippocampus (*F* (1, 12) = 0.26, *p* > 0.05). The cue manipulation was significant decreases in the mPFC (*F* (1, 12) = 14.01, *p* < 0.05) and the hippocampus (*F* (1, 12) = 16.76, *p* < 0.05) compared to the no EE manipulation. The cue showed nonsignificant differences in the NAc (*F* (1, 12) = 2.52, *p* > 0.05) and the amygdala (*F* (1, 12) = 1.59, *p* > 0.05). The interaction of EE and cure reveled the nonsignificant differences in the mPFC (*F* (1, 12) = 1.95, *p* > 0.05), the NAc (*F* (1, 12) = 1.82, *p* > 0.05), the amygdala (*F* (1, 12) = 1.89, *p* > 0.05), and the hippocampus (*F* (1, 12) = 0.27, *p* > 0.05; Table 3).

**Figure 13:**
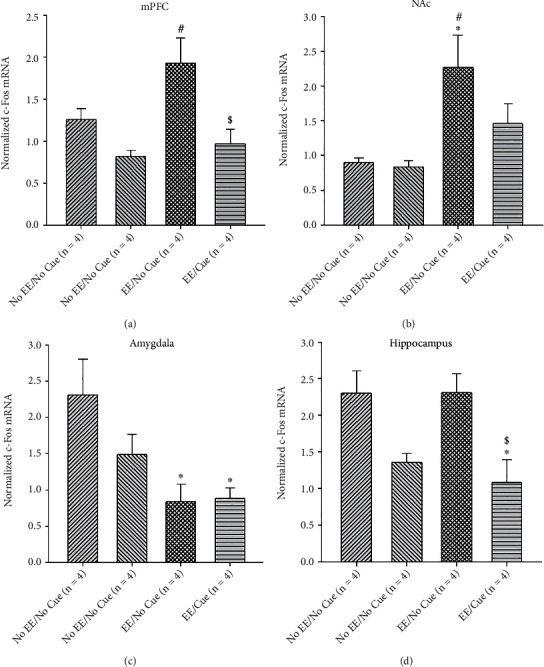
Mean (± SEM) normalized c-Fos mRNA in the mPFC (a), NAc (b), amygdala (c), and hippocampus (d) for the no EE/no cue (*n* = 4), no EE/cue (*n* = 4), EE/no cue (*n* = 4), and EE/cue groups (*n* = 4). EE: environmental enrichment. ^∗^*p* < 0.05 indicates significant differences compared to the no EE/no cue group; #*p* < 0.05 indicates significant differences compared to the no EE/cue group. $*p* < 0.05 indicates significant differences compared to the EE/no cue group.

Furthermore, one-way ANOVA analysis was conducted to analyze the c-Fos mRNA expression in the mPFC, NAc, amygdala, and hippocampus among the no EE/no cue, no EE/cue, EE/no cue, and EE/cue groups. For the mPFC, the factor of group was significant differences (*F* (3, 12) = 6.88, *p* < 0.05). The post hoc Tukey tests indicated that the c-Fos mRNA expression of the EE/no cue group was significant increases than that of the no EE/no cue group (*p* < 0.05); moreover, the c-Fos mRNA expression of the EE/cue group was significantly decreased compared to the EE/no cue group (*p* < 0.05; Figure 13(a)). The result indicated that the EE manipulation is likely to enhance the c-Fos mRNA expression; however, the cue conduction might decrease the c-Fos mRNA expression in the mPFC.

To test the c-Fos mRNA expression of the NAc, one-way ANOVA analysis showed that significant differences occurred in the factor of the group (*F* (3, 12) = 5.85, *p* < 0.05). The post hoc Tukey test showed that the c-Fos mRNA expression of the EE/no cue group was significantly increased compared to that of the no EE/no cue group (*p* < 0.05; Figure 13(b)), indicating that EE manipulations increased the c-Fos mRNA expression in the NAc.

One-way ANOVA was performed to test the amygdala's c-Fos mRNA expression in the EE and cue manipulations. The results showed that the factor of group had significant differences (*F* (3, 12) = 4.82, *p* < 0.05). The post hoc Tukey tests indicated significant decreases in the EE/no cue and the EE/cue groups compared to the no EE/no cue group (*p* < 0.05; Figure 13(c)), indicating that the EE manipulation decreased the c-Fos mRNA expression in the amygdala.

To test c-Fos mRNA of the hippocampus in the EE and cue manipulations, one-way ANOVA analysis was conducted to show that only the EE/cue group was significant decreases in the c-Fos mRNA expression compared to the no EE/no cue and the EE/no cue groups (*p* < 0.05), respectively (Figure 13(d)). Under cue manipulation, the c-Fos mRNA expression of the hippocampus was significantly decreased.

In conclusion, the results of the c-Fos mRNA expression were similar to the findings of the c-Fos protein expression in the mPFC, NAc, amygdala, and the hippocampus (see Tables 2 and 3).

## 4. Discussion

The EE manipulations can reduce footshock-induced fear behavior, and the cue manipulation also reduces fear behavior in PTSD symptoms. The present data showed that the EE is better than the cue manipulation to ameliorate fear behavior in PTSD. The combination of EE and cue induced the most significant reduction effect in fear behavior. Cue manipulation is likely a novel treatment to reduce PTSD symptoms, in particular, to fear behavior.

The c-Fos expression data showed that the Cg1, IL, and NAc upregulated the c-Fos expression in fear behavior; however, the BLA downregulated the c-Fos expression in fear behavior for PTSD symptoms through the EE manipulations, indicating the Cg1, IL, NAc, and BLA controlled the EE-induced fear reduction. In contrast, the Cg1, PrL, IL, CA1, CA3, and DG were downregulated in the c-Fos expression after the cue manipulations in fear behavior, indicating the Cg1, PrL, IL, CA1, CA3, and DG contributed to a cue-induced fear reduction in PTSD. The EE and cue had significant interactions in the Cg1 and NAc for the c-Fos expression. Accordingly, the Cg1 and NAc were involved in the interaction of EE and cue.

The data of the c-Fos mRNA expression were similar to the findings of the c-Fos protein expression. EE manipulations upregulated the c-Fos mRNA expression in the mPFC and the NAc; however, it downregulated the c-Fos mRNA expression in the amygdala. Cue manipulations downregulated the c-Fos mRNA expression in the mPFC and hippocampus. EE and cue interactions did not affect c-Fos mRNA expression in the mPFC, NAc, amygdala, and hippocampus.

### 4.1. Environmental Enrichment, Cue, and Combination of Environmental Enrichment and Cue to Ameliorate Footshock-Induced Fear Behavior in PTSD Symptoms

In the present data, the EE manipulations ameliorated footshock-induced fear behavior. Therefore, the present data of EE is consistent with the previous findings [33]. Dependent on the previous data, a growing body of evidence showed that the EE manipulations decreased fear- and stress-related behaviors in different levels [6–11, 34]. For example, the behavioral test of EE showed that long-term EE suppressed neonatal isolation-induced contextual freezing, but this type of EE did not change single prolonged stress related to anxiety behavior or analgesia. Moreover, EE exposures showed to reduce deep-brain stimulation-induced resistant depression and PTSD in the animal model [6]. Alternatively, the examinations of the brain system in EE ameliorations showed that EE could facilitate the negative feedback regulation of the HPA axis for stress rats, and it ameliorated the abnormal behaviors via downregulation of glucocorticoid receptor expression in the hippocampus and hypothalamus [7]. In another study, activations of serotonin and neuropeptide systems through EE manipulations were shown to reduce anxiety behaviors in the PTSD animal model [11]. Regarding the examination of EE manipulations in neuronal levels, animals with EE exposures increased hippocampal cell proliferation and recovered normal behaviors in PTSD symptoms [34]. Additionally, short-term EE exposures potentiated the extinction process of fear via regulating neuropeptide Y-Y1 receptors in the hippocampus [10]. A previous study has demonstrated that EE exposures ameliorated the extended volumes of the hippocampus and central amygdala caused by traumatic stress [8]. Therefore, the EE manipulations may be effectively nonpharmacological interventions in reducing footshock-induced stress or anxiety in behavior, brain systems, and neuronal levels.

On the other hand, manipulating the cue stimulus is likely a novel approach to examine the amelioration of footshock-induced fear behavior. The present results showed that the cue exposure showed a significant decrease in fear behavior induced by footshock; however, this suppressed effect by the cue stimulus was not higher than that of the EE manipulation. The single cue stimulus may cause the different effects between EE and cue, but the EE is composed of a variety of stimuli, including sensory, physical, social, and cognitive components [12, 13]. Therefore, the EE manipulation is similar to the real contextual stimulus.

### 4.2. Cue and Environmental Enrichment: An Occasion Setter or a CS Associated with the US?

These inconsistent data for the EE and cue manipulations should be explained by that the novelty and salient property of stimulus from the EE exposures might be the crucial factor to ameliorate footshock-induced fear behavior [35, 36]. However, the cue stimulus is short of comprehensive and complete stimuli; instead, the cue exposure only has a single stimulus dimension, causing a weaker novelty and salient property to reduce footshock-induced fear behavior. Interestingly, the combination of EE and cue revealed the highest reduction in footshock-induced fear behavior. What reason is the combination of EE and cue due to the highest reduction for footshock-induced fear behavior? It might be two possibilities. One possible reason is that the cue stimulus is served as a conditioned stimulus (CS) to interact with the unconditioned stimulus (US) to modulate the strengths of the conditioned response (CR) by the rule of classical conditioning [37]. Another possible reason is that the cue stimulus is an occasion setter to enhance the CR, fear behavior. Based on the occasion setting theory, the cue stimulus itself was not involved in the association with the US; however, the occasion setter stimulus only modulates the strength of the CR [38, 39]. According to the present study, the cue stimulus seemingly contributed to the association with the US. Therefore, whether the cue stimulus is an occasion setter or a CS remains scrutinized in further studies.

### 4.3. Involvements of the mPFC, Amygdala, Hippocampus, and NAc in Environmental Enrichment, Cue, or the Combination of Environmental Enrichment and Cue in PTSD Fear Symptoms

To date, no research comprehensively examined how the mPFC, amygdala, and hippocampus contributed to suppressing EE or cue in PTSD symptoms and behaviors. However, amount of research has demonstrated that the mPFC's neuronal activity inhibited the amygdala's negative emotional responses, and the activation of the amygdala conveyed negative feedback information with the negative value to transmit into the mPFC, which explained the valences of this information [40]. This neural pathway of the mPFC projecting to the amygdala has been shown to regulate PTSD symptoms [41, 42]; moreover, the present data showed that the mPFC-amygdala pathway was seemingly involved in the suppression of EE or cue to PTSD symptoms. On the other hand, the hippocampus was suggested to modulate spatial learning and configural memory [19, 43], and the hippocampus is essential to process memory contents in the process of consolidation from the short-term memory to the long-term memory [44, 45]. Accordingly, the hippocampus was acquired by the amygdala's information to modulate the formation of the configural memory and spatial learning. This result is likely consistent with the recent findings that the amygdala interacted with the hippocampus to mediate emotional learning and memory and PTSD symptoms [46]. The amygdala's pathway with the hippocampus might be another crucial neural pathway for PTSD symptoms.

In the reduced effect of the EE in PTSD fear behavior, it was shown that the Cg1 and IL (but not the PrL) of the mPFC were increased in the c-Fos expression, and it indicated that after chronic EE exposures, the mPFC's executive and inhibitory functions interfered with the activity of the amygdala's BLA, which presented decreases in the c-Fos expression. Decreased c-Fos expression of the BLA indicated that the BLA inhibited the negative emotional responses following the EE exposures.

Alternatively, the present data showed that the c-Fos expression of the NAc significantly increased, indicating that EE exposures might induce dopamine neurotransmitters secretions in the NAc due to the novelty or saliency effects following EE exposures [36, 47, 48]. This novelty and saliency of EE's property may be the critical point that the EE manipulations could reduce the symptoms of psychiatric disorders (e.g., PTSD and depression) or neurological diseases (Parkinson's disease or Alzheimer's disease) [35]. How did the novelty and saliency of EE affect the symptoms of psychiatric and neurological diseases which remain to be investigated further?

In particular, the cue stimulus manipulations showed different results that the Cg1, PrL, and IL of the mPFC and the CA1, CA3, and DG of the hippocampus decreased the c-Fos expression compared to the EE manipulations. Accordingly, the cue manipulations may play a different role in the reduction of footshock-induced fear behavior when compared to the EE manipulations. The cue stimulus is not contextual, and it is likely an occasion setting to modulate fear behavior induced by footshock in the PTSD animal model [39, 49]. Thus, the occasion setter role of the cue fully decreased the c-Fos expression in the whole subregions of the mPFC and the hippocampus. The results indicated that the cue stimulus interfered with the executive and inhibitory functions resulted in the mPFC dysfunctions; moreover, the cue stimulus decreased the c-Fos expression in the CA1, CA3, and DG of the hippocampus, indicating the hippocampus revealed dysfunctions in spatial learning and configural memory. Accordingly, the c-Fos data of the cue stimulus manipulations revealed a worse reduction to footshock-induced fear behavior than those of the EE manipulation and the combination of the EE and cue manipulations.

In the examinations of the interaction between EE and cue, the results showed that significant differences occurred in the Cg1 of the mPFC and the NAc to reduce footshock-induced fear behavior. Why did the c-Fos expression of the Cg1 and NAc produce an interaction between the EE and cue manipulations? It is due to the property of the Cg1 governing the stress or anxiety behaviors and the property of the NAc involving the reward or reinforcement process. This emerged issue should be concerned with further studies.

## 5. Conclusion

The EE and cue suppressed footshock-induced fear behavior; however, the EE manipulation produced a more potent suppression in footshock-induced fear behavior compared to the cue manipulation; although, the cue stimulus also reduced footshock-induced fear behavior. These data supported the previous findings [6, 8, 11, 35, 36]. For example, EE exposures reduced traumatic stress and increased the volume of the hippocampus and central amygdala [8]; moreover, it ameliorated anxiety [11], depression [6], PTSD symptoms [6], and footshock-induced fear behaviors [35, 36] in the animal model. Notably, the combination of EE and cue manipulations produced the strongest reduction in footshock-induced fear behavior. Under the EE manipulations, c-Fos upregulation occurred in the Cg1, IL, and NAc, but c-Fos downregulation occurred in the BLA. Under cue manipulations, c-Fos downregulation occurred in the Cg1, PrL, IL, CA1, CA3, and DG. The data of the c-Fos mRNA expression were similar with the data of the c-Fos protein expression. The c-Fos mRNA expression was upregulation in the mPFC and amygdala in the EE manipulations; the amygdala was downregulated for the c-Fos mRNA expression in the EE manipulations. Cue manipulations were decreases in the c-Fos mRNA expression for the mPFC and hippocampus. The present findings are likely to offer contributions for novel and nonpharmacological treatments to PTSD symptoms. The present data might help understand the amelioration mechanisms of PTSD fear symptoms in the brain.

## Figures and Tables

**Table 1:** Mean (± SEM) freezing time (sec.) was analyzed by a 2 × 2 × 3 three-way mixed analysis of variance (ANOVA) (environmental enrichment vs. cue vs. session).

2 × 3 × 2 three-way mixed ANOVA	
Environmental enrichment	*F*_1,20_ = 70.73, *p* < 0.05^∗^
Cue	*F*_1,20_ = 14.14, *p* < 0.05^∗^
Session	*F*_2,40_ =27.95, *p* < 0.05^∗^
Environmental enrichment × cue	*F*_1,20_ = 3.25, *p* > 0.05
Environmental enrichment × session	*F*_2,40_ = 2.11, *p* > 0.05
Cue × session	*F*_2,40_ = 0.28, *p* > 0.05
Environmental enrichment × cue × session	*F*_2,40_ = 0.89, *p* > 0.05

^∗^ *p* < 0.05, significant difference.

**Table 2:** Effects of environmental enrichment and cue for PTSD in the c-Fos expression for selected brain areas in the situational reminder phase.

	Cg1	PrL	IL	NAc	BLA	CA1	CA3	DG
Environmental enrichment	↑	—	↑	↑	↓	—	—	—
Cue	↓	↓	↓	—	—	↓	↓	↓
Interaction of Cue and environmental enrichment	+	—	—	+	—	—	—	—

Note: (↑): increases; (↓): decreases; (-): nonsignificant difference; (+): *p* < 0.05.

**Table 3:** Effects of environmental enrichment and cue for PTSD in the normalized c-Fos mRNA expression for selected brain areas in the situational reminder phase.

	mPFC	NAc	Amygdala	Hippocampus
Environmental enrichment	↑	↑	↓	—
Cue	↓	—	—	↓
Interaction of Cue and environmental enrichment	—	—	—	—

Note: (↑): increases; (↓): decreases; (-): nonsignificant difference; (+): *p* < 0.05.

## Data Availability

The raw data can be accessed through the following link: https://www.dropbox.com/sh/r11cj1scrvggykz/AAC__MvtjwTIMAAQYb9p92p0a?dl=0.
